# Comprehensive Laboratory Analysis of a Scrub Typhus and H1N1 Influenza Co-Infection: A Case Report from Hainan, China

**DOI:** 10.3390/pathogens14080810

**Published:** 2025-08-15

**Authors:** Siqi Chen, Fahui Wang, Shannan Wu, Yuanze Chen, Yi Niu, Yijia Guo, Dachuan Lin, Xiuji Cui, Ruoyan Peng, Zhao Xu, Biao Wu, Min Liao, Yongguo Du, Liyuan Zhang, Feifei Yin

**Affiliations:** 1 The University of Hong Kong Joint Laboratory of Tropical Infectious Diseases—Hainan Medical University, Key Laboratory of Tropical Translational Medicine of Ministry of Education, Academician Workstation of Hainan Province, School of Basic Medicine and Life Sciences, Hainan Medical University, Haikou 571199, China; qisi49u@muhn.edu.cn (S.C.); hypd00021@muhn.edu.cn (Z.X.); 2 Department of Respiratory Medicine, The Second Affiliated Hospital, Hainan Medical University, Haikou 570311, China; 3 Animal and Plant Quarantine Center, Haikou Customs District, Haikou 570311, China; wsnhiciq@126.com; 4 Department of Infectious Diseases, The Second Affiliated Hospital, Hainan Medical University, Haikou 570311, China; cyuanze@muhn.edu.cn (Y.C.);; 5 Hainan General Hospital, Hainan Affiliated Hospital of Hainan Medical University, Haikou 570100, China

**Keywords:** scrub typhus, influenza A (H1N1), co-infection, real-time PCR, next-generation sequencing (NGS)

## Abstract

Co-infection of *Orientia tsutsugamushi* and influenza A virus complicates diagnosis and treatment in endemic regions because of overlapping clinical features and potential synergistic inflammation. We describe a 68-year-old woman from Hainan, China, who presented with five days of high fever (39.2 °C), nonproductive cough, eschar formation, lymphadenopathy, cytopenias, elevated liver enzymes, and raised inflammatory markers. On the day of admission, influenza A was confirmed by rapid antigen test and *Orientia tsutsugamushi* IgM/IgG was detected via colloidal-gold immunochromatography, prompting concurrent oseltamivir and doxycycline therapy. Quantitative PCR on day 2 measured an *Orientia tsutsugamushi* load of 2.85 × 10^4^ copies/mL (Cq 28.86), and targeted next-generation sequencing on day 3 revealed a high H1N1pdm09 viral burden (>1 × 10^6^ copies/mL) with low-level human herpesvirus 1 co-detection. Nested PCR and Sanger sequencing assigned *Orientia tsutsugamushi* to the Karp_A lineage and influenza A to clade 6B.1A.5a.2a. The patient defervesced by hospital day 2, laboratory indices normalized by day 3, and radiographic abnormalities resolved by day 6. This first documented *Orientia tsutsugamushi*–influenza A co-infection in China highlights the value of integrating rapid serology, qPCR quantification, nested PCR genotyping, and tNGS for early, precise dual-pathogen identification. Systematic multi-pathogen screening during overlapping transmission seasons is recommended to guide timely combination therapy and enhance epidemiological surveillance.

## 1. Introduction

Scrub typhus, caused by the obligate intracellular bacterium *Orientia tsutsugamushi*, is a neglected tropical disease historically endemic to the “tsutsugamushi triangle” in the Asia–Pacific region [1,2,3]. Recent studies indicate an increasing geographic distribution and rising incidence of scrub typhus across the traditional endemic Asia–Pacific region, as well as sporadic occurrences beyond previously recognized boundaries, suggesting an evolving epidemiological landscape [3,4]. Scrub typhus is often under-recognized despite its high incidence and morbidity in endemic areas. Clinical manifestations of Scrub typhus vary from mild symptoms like fever and headache to severe complications such as pneumonia, acute respiratory distress syndrome (ARDS), meningoencephalitis, acute kidney injury, or disseminated intravascular coagulation [5]. Mortality reports vary widely around a median mortality of 6.0% for untreated and 1.4% for treated Scrub typhus [6]. The substantial genotypic heterogeneity of *Orientia tsutsugamushi* further complicates clinical management and the development of protective immunity, highlighting the need for precise pathogen characterization [7].

Concurrent circulation of influenza A virus, particularly subtype H1N1, adds additional complexity in clinical diagnosis and patient management due to overlapping clinical presentations with Scrub typhus. Co-infection with *Orientia tsutsugamushi* and H1N1 influenza has emerged as a significant concern in endemic regions, potentially amplifying inflammatory responses and complicating disease progression. However, current diagnostic methods for scrub typhus still have significant limitations. The indirect immunofluorescence assay (IFA), considered the diagnostic gold standard due to its high specificity, is not widely available in routine clinical settings and often yields delayed results. Enzyme-linked immunosorbent assay (ELISA) and rapid tests such as colloidal-gold immunochromatography are easier to perform and allow quicker decision-making. Still, their sensitivity can vary, especially in early infection. Molecular approaches, including quantitative PCR and nested PCR targeting the *tsa56* gene, offer greater accuracy in the acute phase and allow both pathogen quantification and genotyping. Given these constraints, combining multiple diagnostic techniques is often necessary to confirm scrub typhus and guide appropriate treatment [8,9].

This study addresses the critical gap in comprehensive, quantitative, and genotypic diagnostic approaches for co-infections of *Orientia tsutsugamushi* and H1N1. We describe the clinical, laboratory, and molecular profile of a scrub typhus and H1N1 co-infection case from Hainan, China, to illustrate how such integrated approaches can improve diagnostic accuracy, inform individualized treatment decisions, and strengthen surveillance strategies in regions where multiple pathogens co-circulate.

## 2. Case Presentation

A 68-year-old female rubber tapper from Chengmai County, Hainan Province, was admitted to the Department of Respiratory Medicine, The Second Affiliated Hospital, Hainan Medical University, on 21 June 2024, with a five-day history of intermittent high fever (peak 39 °C), chills, dizziness, and non-productive cough. She denied gastrointestinal and urinary symptoms. Her medical history was generally unremarkable; however, she had sought symptomatic treatment, including antipyretics and empirical antibiotics, at a local clinic shortly after symptom onset. Specific details of the medications administered were unavailable. The patient reported no known exposure to individuals diagnosed with influenza or scrub typhus.

**Figure 1:**
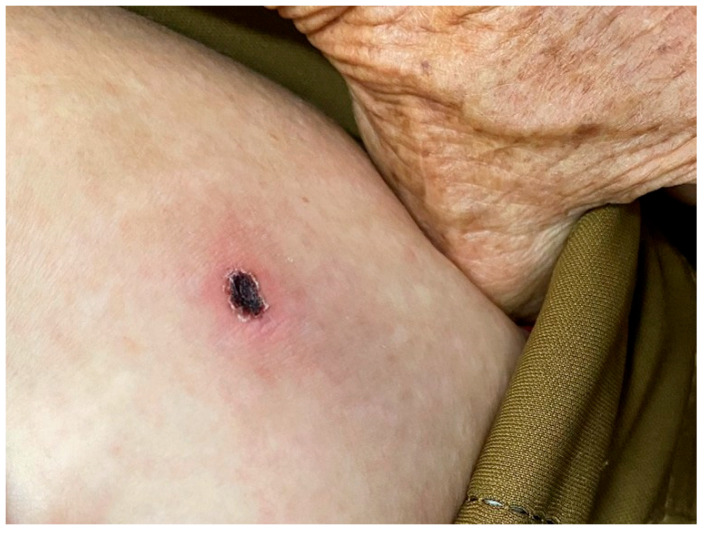
Physical examination revealed a 3 × 2.5 cm eschar surrounded by erythema on the left mid-abdomen.

On admission, her vital signs were notable for a temperature of 39.4 °C, pulse rate of 88 beats/min, respiratory rate of 20 breaths/min, and blood pressure of 133/77 mmHg. Physical examination revealed a 3 × 2.5 cm eschar surrounded by erythema on the left mid-abdomen, along with non-tender lymphadenopathy palpated in the axillary and inguinal regions (Figure 1). No other rash or focal neurological deficits were identified.

**Figure 2:**
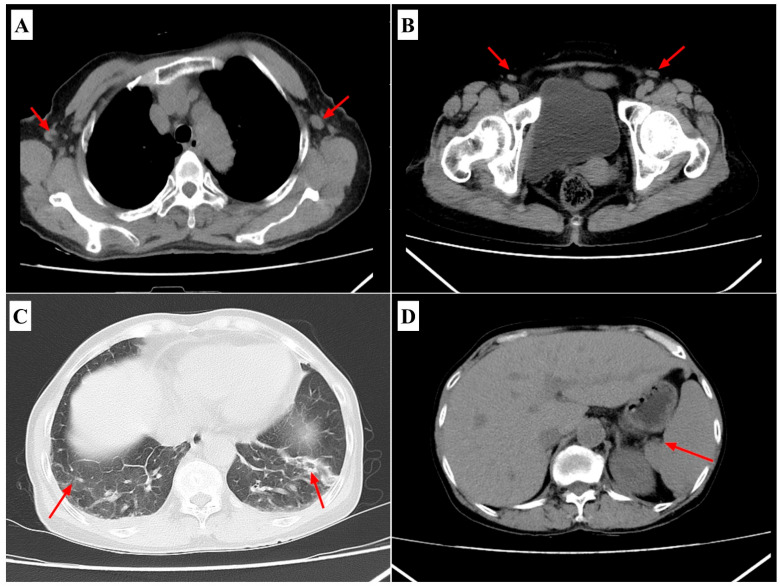
CT scan of the chest demonstrates. (**A**) Axillary lymphadenopathy; (**B**) inguinal lymphadenopathy; (**C**) bilateral multiple pulmonary inflammatory lesions, predominantly in the lower lobes; (**D**) splenomegaly. (The red arrow in the figure indicates the lesion site).

Laboratory investigations on admission showed leukopenia (5.83 × 10^9^/L; reference range 4.0–11.0 × 10^9^/L) with neutrophil predominance (76.2 %; reference range: 40–75%), thrombocytopenia (72 × 10^9^/L; reference range 150–400 × 10^9^/L), elevated liver enzymes (ALT 323 U/L; reference range < 41 U/L; AST 355 U/L; reference range < 40 U/L), hypoalbuminemia (27.1 g/L; reference range 35–52 g/L), hyponatremia (126.9 mmol/L; reference range 135–145 mmol/L), and markedly elevated inflammatory markers (hs-CRP 103.2 mg/L; reference range < 5 mg/L; IL-6 121 pg/mL; reference range < 7 pg/mL; PCT 1.31 ng/mL; reference range < 0.1 ng/mL). Coagulation tests indicated prolonged activated partial thromboplastin time (62.8 s; reference range 25–35 s) and elevated D-dimer levels (20.62 μg/mL; reference range < 0.5 μg/mL) (Table 1). Chest computed tomography indicated bilateral lower-lobe infiltrates, axillary lymphadenopathy, and splenomegaly (Figure 2). Abdominal ultrasonography confirmed mild hepatosplenomegaly without signs of biliary obstruction.

Given the clinical presentation suggestive of influenza-like illness, rapid antigen testing on the first day immediately confirmed influenza A infection, and colloidal-gold immunochromatography simultaneously detected *Orientia tsutsugamushi*-specific IgM and IgG antibodies. This point-of-care assay yields qualitative (positive/negative) results; therefore, quantitative antibody titers were not available at admission. Standard aerobic and anaerobic blood cultures were also obtained upon admission and incubated for five days, revealing no bacterial or fungal growth. PCR assays targeting other pathogens with similar clinical presentations, including other Rickettsia species, dengue virus, *Leptospira* spp., and *Plasmodium* spp. (malaria), were performed and yielded negative results. As both pathogens were identified upon admission, antiviral therapy with oseltamivir and antibacterial treatment with doxycycline were initiated promptly. On the second day of hospitalization, quantitative real-time PCR (qPCR) confirmed the presence of *Orientia tsutsugamushi*, quantifying the pathogen load at 2.85 × 10^4^ copies/mL. On day 3, targeted next-generation sequencing (tNGS) results identified a high viral load of influenza A H1N1pdm09 (>1.0 × 10^6^ copies/mL) and a low-level presence of herpes simplex virus 1 (approximately 2.0 × 10^3^ copies/mL). This respiratory panel detects 153 common respiratory pathogens and enables simultaneous viral and bacterial screening. Nested PCR and subsequent Sanger sequencing further characterized the pathogens, assigning *Orientia tsutsugamushi* to genotype Karp_A and influenza A virus to clade 6B.1A.5a.2a. Taken together, the near-contemporaneous detection of high pathogen loads—*Orientia tsutsugamushi* by qPCR on day 2 and H1N1pdm09 by targeted NGS on day 3—supports active co-infection during the acute phase; however, in the absence of quantitative anti-body titers, the precise temporal sequence of the two infections cannot be inferred.

The patient was treated with oral doxycycline (100 mg every 12 h) and oseltamivir (75 mg every 12 h) for seven days, accompanied by supportive therapy. Fever resolved within two days, and laboratory parameters substantially improved by the third day of hospitalization. Follow-up imaging on day six showed resolution of pulmonary infiltrates and lymphadenopathy. The patient was discharged on day seven in stable condition and remained asymptomatic at two-week follow-up.

## 3. Laboratory Studies and Evolutionary Analysis

### 3.1. Sample Collection and Nucleic Acid Extraction

Whole-blood and nasopharyngeal swab specimens were collected at admission into EDTA tubes and viral transport medium, respectively. DNA was extracted from 200 µL whole blood using the QIAamp DNA Mini Kit (Qiagen, Hilden, Germany) and eluted in 50 µL buffer. Viral RNA was extracted from 140 µL swab fluid with the QIAamp Viral RNA Mini Kit (Qiagen, Hilden, Germany) and eluted in 60 µL buffer.

### 3.2. Detection and Genetic Analysis of Orientia tsutsugamushi

Initial rapid screening for *Orientia tsutsugamushi* was performed using a qualitative colloidal-gold lateral-flow immunochromatographic assay (Wantai, Beijing, China) detecting pathogen-specific IgM and IgG. Positive results prompted subsequent qPCR targeting the tsa56 gene, performed according to previously established protocols by our group [10]. The patient’s sample yielded a quantification cycle (Cq) of 28.86, corresponding to a bacterial load of 2.853 × 10^4^ copies/mL. As a rapid adjunct at admission, colloidal-gold immunochromatography provided qualitative evidence of *Orientia tsutsugamushi* IgM/IgG but does not report titers. IFA/ELISA titration (including paired sera) was not performed at admission due to turnaround and availability constraints, and subsequent molecular testing—qPCR and nested PCR targeting the tsa56 gene—was therefore prioritized to confirm active infection and enable genotyping.

**Figure 3:**
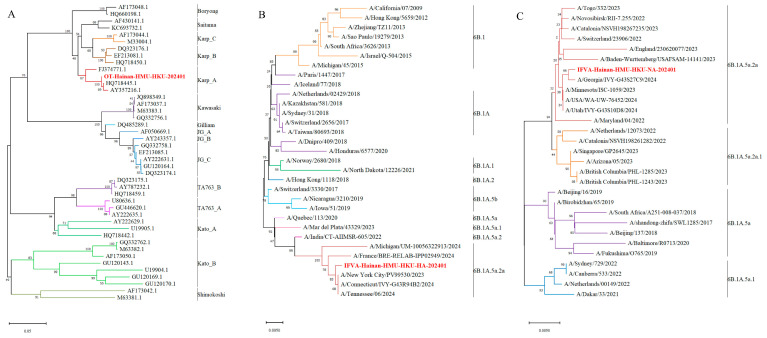
Phylogenetic tree based on the tsa56 gene of *Orientia tsutsugamushi* and the HA and NA genes of influenza A. (**A**) Phylogenetic tree based on the tsa56 gene of *Orientia tsutsugamushi*; (**B**) phylogenetic tree based on the HA gene of influenza A virus; (**C**) phylogenetic tree based on the NA gene of influenza A virus.

For genotyping, a 483 bp fragment of the tsa56 gene was amplified by nested PCR and subsequently subjected to Sanger sequencing as previously described [10,11]. The nested PCR assay was performed using the following primers: for the first-round PCR, forward primer 5′-TCAAGCTTATTGCTAGTGCAATGTCTGC-3′ and reverse primer 5′-AGGGATCCCTGCTGCTGTGCTTGCTGCG-3′; for the second-round PCR, forward primer 5′-GATCAAGCTTCCTCAGCCTACTATAATGCC-3′ and reverse primer 5′-CTAGGGATCCCGACAGATGCACTATTAGGC-3′. The analytical sensitivity of this nested PCR assay was approximately 10 copies/reaction. The resulting sequences were compared with reference strains in the NCBI GenBank database using BLAST (version 2.15.1; National Center for Biotechnology Information, Bethesda, MD, USA). Phylogenetic analysis was conducted using MEGA-X (version 11.0.11; MEGA Software, State Collage, PA, USA) with the Neighbor-Joining algorithm and 1000 bootstrap replicates. The isolate from this study, designated OT-Hainan-HMU-HKU-202401 (accession no. PV359176), clustered within the Karp_A genotype and exhibited 100% nucleotide identity with a previously identified Hainan isolate from 2021 (accession no. PV633745) (Figure 3A).

### 3.3. Detection and Genetic Analysis of Influenza A Virus

Throat swab specimens were first tested by rapid antigen assay immediately upon admission, confirming influenza A virus infection. The samples were further analyzed using targeted next-generation sequencing (tNGS; KingMed Diagnostics, China), employing a comprehensive panel covering conserved genomic regions from 153 respiratory pathogens. The analysis quantified influenza A H1N1pdm09 viral load at >1.0 × 10^6^ copies/mL, alongside low-level human herpesvirus 1 (~2.0 × 10^3^ copies/mL). The patient exhibited no mucocutaneous lesions, neurological signs, or other features suggestive of HSV disease during hospitalization, and no radiographic or laboratory evidence indicated HSV-related organ involvement; therefore, antiviral therapy targeting HSV was not initiated and HSV was not included in the final diagnosis.

For further subtype confirmation, nested RT-PCR amplified the approximately 1300 bp HA gene and 1100 bp NA gene regions, followed by Sanger sequencing [12]. Phylogenetic analyses conducted using the maximum likelihood approach in MEGA-X included reference sequences from NCBI and GISAID. The H1N1 HA and NA sequences grouped within the global 6B.1A.5a.2a lineage, confirming ongoing circulation of this subclade in Hainan (Figure 3B,C).

## 4. Discussion and Conclusions

The co-infection of *Orientia tsutsugamushi* and influenza A virus (H1N1pdm09) presents a unique diagnostic and therapeutic challenge in endemic settings because of overlapping clinical manifestations. This report details the first documented co-infection case of *Orientia tsutsugamushi* and influenza A virus subtype H1N1pdm09 in China, highlighting the diagnostic challenges and the necessity of prompt, accurate pathogen identification. Delayed initiation of doxycycline therapy in scrub typhus is associated with increased mortality rates, whereas the therapeutic efficacy of oseltamivir declines significantly when administered beyond 48 h following influenza symptom onset [13,14,15]. In this case, the employment of rapid antigen testing for influenza A alongside colloidal-gold immunochromatography for *Orientia tsutsugamushi* on the day of admission enabled the immediate initiation of oseltamivir and doxycycline, respectively. Early confirmation of *Orientia tsutsugamushi* by quantitative PCR (qPCR) on hospital day two provided a precise measure of bacterial burden (2.85 × 10^4^ copies/mL), while tNGS on day three quantified influenza viral load (>1.0 ×10^6^ copies/mL) and detected low-level human herpesvirus 1 co-infection. This stepwise, multimodal approach exemplifies how ultra-early pathogen identification can guide timely, tailored therapy.

Quantitative pathogen load measurement and genotypic characterization were instrumental in refining clinical management and informing regional surveillance [10,11,16]. In this study, qPCR revealed an *Orientia tsutsugamushi* load of 2.85 × 10^4^ copies/mL (Cq 28.86), considerably higher than the median of 3.11 × 10^3^ copies/mL reported in a recent Hainan cohort, which may reflect infection by a high-virulence Karp_A genotype or influenza-induced impairment of host antibacterial defenses [10]. Influenza A–induced dysregulation of innate immunity and epithelial barrier dysfunction have been shown to facilitate secondary bacterial proliferation, potentially accounting for the elevated *Orientia tsutsugamushi* burden [17,18]. The *Orientia tsutsugamushi* isolate was assigned to the Karp_A genotype, consistent with prior reports from Hainan, and the influenza A virus clustered within the local circulating 6B.1A.5a.2a clade. Karp strains have been associated with heightened TNF-α and IL-6 responses and greater organ injury in experimental models, raising the possibility that Karp_A–H1N1 co-infection may amplify systemic inflammation [5,19].

Previous reports have described five additional instances of *Orientia tsutsugamushi*–influenza A co-infection: the first documented case in Korea (2011), a 4.7% co-infection rate (3/64) among H1N1 inpatients in India (2020) [20], and the first Japanese case (2025) [21]. In all instances, patients presented with fever, cough, and myalgia; eschars were observed in two-thirds of cases, and transaminase elevations suggested hepatic involvement. Radiographically, bilateral pulmonary infiltrates and multifocal inflammatory lesions were common, and several patients developed dyspnea or respiratory failure necessitating mechanical ventilation—findings consistent with synergistic pathogen interactions [22]. These data emphasize the inadequacy of relying on clinical signs alone and underscore the importance of systematic, multimodal molecular screening for febrile patients with atypical presentations. Recent epidemiological data indicate a rising incidence of scrub typhus across endemic regions, including Hainan Province [23,24]. Globally, scrub typhus threatens over 1 billion people and causes at least 1 million clinical cases annually, underscoring the growing public health burden and the need for strengthened surveillance in endemic provinces such as Hainan [1,6]. In China, national surveillance documented a >16-fold rise in incidence from 0.09 per 100,000 in 2006 to 1.60 per 100,000 in 2016, alongside geographic expansion to all 31 provinces, spanning both rural and urban settings [23]. In Hainan Province specifically, 3260 scrub typhus cases were reported during 2011–2020 with year-on-year increases and dual seasonal peaks; farmers and adults aged 50–59 years constituted the highest-risk groups [24]. Furthermore, in addition to influenza A, co-infections of *Orientia tsutsugamushi* with other pathogens—such as *Mycoplasma pneumoniae*, *Leptospira* spp., severe fever with thrombocytopenia syndrome virus, and dengue virus—have been increasingly reported [7,25,26], often leading to more severe clinical presentations and complicating diagnosis and management. These observations underscore the importance of comprehensive, multi-pathogen screening in febrile patients in endemic settings.

In the present case, serological assays, qPCR quantification, nested PCR genotyping, and tNGS were performed within the first three days of admission, enabling definitive, quantitative dual-pathogen identification. Although conventional diagnostics could guide initial treatment decisions, the integration of tNGS and genotyping provided precise pathogen quantification, identified clinically relevant co-infections, and clarified local epidemiological contexts, thereby enhancing both patient management and regional surveillance. Adoption of such standardized workflows in endemic settings is advocated to improve co-infection detection, inform prompt dual-pathogen therapy, and enhance epidemiological surveillance.

Despite successful clinical outcomes in this case, several questions remain. The prognostic value of dual pathogen load dynamics and potential immunological interactions between *Orientia tsutsugamushi* and influenza virus warrant further investigation in larger cohorts. Standardized protocols should be developed to validate cutoffs for qPCR-derived bacterial loads and to quantify their relationship with disease severity and treatment response. Another important limitation is that blood-based PCR assays for scrub typhus typically exhibit lower sensitivity compared to PCR performed on eschar biopsy specimens, due to higher bacterial loads present in eschars. However, eschar specimens were not obtained in this case owing to practical constraints. Future studies should prioritize obtaining eschar biopsies whenever feasible to enhance diagnostic accuracy. Additionally, the absence of quantitative serologic titers or paired IFA in this report limits our ability to precisely determine infection timing. Incorporating paired serology and serial qPCR assays into future studies will help clarify the chronology of dual infections more accurately.

In conclusion, the earliest possible implementation of combined rapid serology, qPCR, and tNGS facilitated accurate, quantitative, and genotypic diagnosis of *Orientia tsutsugamushi*–influenza A co-infection, enabling prompt dual-pathogen therapy and favorable patient recovery. Adoption of such integrative molecular workflows in endemic regions is recommended to improve clinical outcomes and to support comprehensive infectious disease surveillance.

## Figures and Tables

**Table 1:** Patient-related laboratory test results. (comparison of admission and discharge results, ↑ indicates that the patient’s test indicators are higher than the highest normal reference value, ↓ indicates that they are lower than the lowest normal reference value).

Category	22-June Treatment/Peak Phase	26-June Post-Treatment
Body temperature (°C)	39.4 ↑	37
hs-CRP (mg/L)	103.230 ↑	9.150
IL-6 (pg/mL)	121.00 ↑	Normal
PCT (ng/mL)	1.31 ↑	0.18 ↑
Neutrophils Percentage	76.2% ↑	32.0% ↓
Lymphocytes Percentage	15.4% ↓	58.9% ↑
Platelet Count (×103/mL)	72 ↓	Normal
AST (U/L)	355 ↑	70 ↑
ALT (U/L)	323 ↑	142 ↑
TP (g/L)	47.5 ↓	56.4 ↓
ALB (g/L)	27.1 ↓	30.6 ↓
A/G Ratio	1.33	1.19 ↓
TBIL (μmol/L)	19.6 ↑	Normal
DBIL (μmol/L)	12.7 ↑	Normal
APTT (s)	62.8 ↑	Normal
D-Dimer (μg/mL)	20.62 ↑	1.68 ↑

hs-CRP, High-Sensitivity C-Reactive Protein; IL-6, Interleukin-6; PCT, Procalcitonin; AST, Aspartate Aminotransferase; ALT, Alanine Aminotransferase; TP, Total Protein; ALB, Albumin; A/G Ratio, Albumin-to-Globulin Ratio; TBIL, Total Bilirubin; DBIL, Direct Bilirubin; APTT, Activated Partial Thromboplastin Time.

## Data Availability

All sequences analyzed during this study are available from the NCBI database (GenBank accession no. PP994860, PP004861, PV359176).
